# Effects of Hypertension Alone and in Comorbidity with Diabetes on Death within 30 Days among Inpatients with COVID-19 Infection

**DOI:** 10.34172/jrhs.2022.100

**Published:** 2022-12-29

**Authors:** Erfan Ayubi, Fatemeh Torkaman Asadi, Shiva Borzouei, Behnaz Alafchi, Mobin Faghih Soleimani, Saman Khosronejad, Salman Khazaei, Seyed Saman Talebi

**Affiliations:** ^1^Social Determinants of Health Research Center, Hamadan University of Medical Sciences, Hamadan, Iran; ^2^Department of Infectious Disease, School of Medicine, Hamadan University of Medical Sciences, Hamadan, Iran; ^3^Infectious Disease Research Center, Hamadan University of Medical Sciences, Hamadan, Iran; ^4^Department of Internal Medicine, School of Medicine, Hamadan University of Medical Sciences, Hamadan, Iran; ^5^Modeling Noncommunicable Diseases Research Center, Hamadan University of Medical Sciences, Hamadan, Iran; ^6^Student Research Committee, Hamadan University of Medical Sciences, Hamadan, Iran; ^7^Department of Epidemiology, School of Public Health, Hamadan University of Medical Sciences, Hamadan, Iran; ^8^Research Center for Health Sciences, Hamadan University of Medical Sciences, Hamadan, Iran

**Keywords:** COVID-19, Comorbidities, Hypertension, Diabetes, Mortality

## Abstract

**Background:** Hypertension and diabetes are common comorbidities in patients with COVID-19 and could be influencing the mortality of such patients. The present study aimed to evaluate the effects of hypertension alone and in comorbidity with diabetes on the death within 30 days among inpatients with COVID-19 in presence of well-known determinates of COVID-19 death.

**Study Design:** A case-control study.

**Methods:** Four groups of COVID-19 inpatients including controls, diabetes alone, hypertension alone, and hypertension and diabetes comorbidities were defined. Each study groups did not have underlying diseases other than hypertension and diabetes. Demographic and general characteristics, underlying diseases, and hospital course events were extracted from medical records. The outcome of interest was alive at discharge/ death within 30 days after admission. Multivariable binary logistic analysis was employed to estimate the effect measures.

**Results:** The number of death within 30 days among controls (n=1359), diabetes alone (159), hypertension alone (406) and hypertension and diabetes comorbidities (188) were 12.68%, 15.72%, 20.74% and 26.74%, respectively. According to three multivariable analyses after adjusting older age, hospital length of stay, and intensive care unit (ICU) admission separately, the odds of death within 30 days in COVID-19 patients with having hypertension and diabetes comorbidities was 1.58, 2.13 and 1.91 times of patients without such comorbidities, respectively (*P*<0.015). The effect of hypertension alone was also significant after adjusting hospital length of stay and ICU admission but not for older age.

**Conclusion:** Our results suggest that comorbidities, such as hypertension and diabetes may be associated with COVID-19-related deaths independent of other underlying diseases, older age, and adverse hospital course events.

## Background

 Since the beginning of the COVID-19 pandemic, about 636 million patients and more than 6.5 million deaths worldwide were attributed to this virus until 18 November 2022.^1^ Most of the COVID-19 patients in the acute phase of infection have a favorable clinical outcome, but the risk of severe forms of COVID-19 and poor outcomes increases significantly with increasing age, and the simultaneous presence of comorbidities as well as chronic diseases.^2,3^

 Hypertension and type 2 diabetes are most frequent comorbidities among patients with COVID-19 infection.^4,5^ Although association between the deaths related COVID-19 and hypertension has suggested in previous studies.^6-9^ However, there are some controversies about effect of hypertension on mortality of COVID-19 patients. One study suggests the hypertension only affect severity of COVID-19 infection and not death or acute respiratory distress syndrome (ARDS)/respiratory failure.^10^ Another study showed hypertension was associated with poor composite outcome including death, intensive care unit (ICU) admission and ARDS.^11^ Previous study indicate there was no difference between non-hypertension and hypertension groups in regard to 28-day mortality and 60-day mortality rates.^12^

 As suggested before independent effect of hypertension on mortality alone of patients with COVID-19 infection needs more clarification.^13,14^ Interaction between hypertension and other comorbidities have demonstrated as risk factors for mortality among COVID-19 patients. For example, previous studies have highlighted the importance of co-morbidities hypertension and type 2 diabetes mellitus as a risk factor for death among patients with COVID-19 infection.^10,15^ However, the effect of hypertension and diabetes alone or in combination with each other on the death due to COVID-19 still can be more clarified.^16^

 To extend previous knowledge, the present study aimed to evaluate the effects of hypertension alone and in comorbidity with diabetes on death within 30 days among inpatients with COVID-19 with considering well-known determinants including older age, underlying diseases, hospital length of stay and admission to ICU.

## Methods

###  Study design and patients

 The current study was retrospective study design that was conducted on patients with COVID-19 who were admitted to Sina (Farshchian) and Shahid Beheshti hospitals in Hamadan between March 2020 and June 2021. The inclusion criteria were inpatients with diagnosis of COVID-19 based on polymerase chain reaction (PCR) test.

 In this study we categorized patients to the four groups: group (1) patients without hypertension and diabetes, as control group (2) those with diabetes patients without hypertension, group (3) those with hypertension and without diabetes and group (4) those with hypertension and diabetes comorbidities. In all 4 groups, there were no patients with underlying chronic diseases including neoplasms, heart diseases, chronic respiratory disease, chronic kidney disease, neurological disease and immunosuppression. The outcome of interest was alive at discharge/death within 30 days after admission. Patients whose outcome was unknown were excluded from the study. The present study was approved by the Ethics Committee of Hamadan University of Medical Sciences.

###  Data collection 

 In order to collect information of patients from their medical files, a researcher-made checklist was used, and this checklist contains the following information: (a) Demographic and general characteristics: gender, age, weight, height, body mass index (BMI), location, occupation and marital status, (b) Underlying diseases such as: hypertension and diabetes. (c) Hospital course events information: duration of length stay, admission to the ICU, outcome of lived and death at discharge.

###  Statistical analysis

 Demographics and clinical characteristics according the three study groups described as number (%) and were tested using chi square tests. Univariate and multivariable binary logistic regression analysis was used to estimate odds ratios (OR) and 95% confidence interval (CI). In multivariable analysis, several scenarios were evaluated to estimate independent effects of hypertension alone and in comorbidity with diabetes after adjusting important and well-known determinant of COVID-19 deaths. Statistical significance was set as *P ≤*0.05. All statistical analyses were performed using Stata version 14.

## Results

 Altogether, 1359 controls, 159 cases with diabetes, 406 with hypertension and 188 with hypertension and diabetes comorbidities were selected for the study. Table 1 shows the general characteristics and hospital course events of these subjects. The distribution of sex, age group, marital status, occupation, BMI, hospital length of stay and ICU admission were different across the studied group (*P* < 0.05), however, there was no statistically difference in location and smoking status.

**Table 1:** General characteristics and hospital course events according to the study groups

**Characteristics **	**Control ** **(n=1359)**		**Diabetes ** **(n=159)**		**Hypertension** **(n=406)**		**Hypertension+diabetes** **(n=188)**		***P*** **-value**
	**Number**	**Percent**	**Number**	**Percent**	**Number**	**Percent**	**Number**	**Percent**	
Gender									0.001
Female	541	39.81	77	48.43	229	56.40	124	65.96	
Male	818	60.19	82	51.57	177	43.60	64	34.04	
Age (y)									0.001
< 50	654	48.19	41	26.11	43	10.64	18	9.57	
≥ 50	703	51.81	116	73.89	361	89.36	170	90.43	
Location									0.503
City	1145	84.32	136	85.53	333	82.02	153	81.38	
Village	213	15.68	23	14.47	73	17.98	35	18.62	
Marital status									0.001
Married	1216	89.48	143	89.94	328	80.79	152	81.28	
Single, divorced, widow	143	10.52	16	10.06	78	19.21	35	18.72	
Occupation									0.001
Employed	468	56.25	69	65.71	214	79.26	107	82.95	
Unemployed	364	43.75	36	34.29	56	20.74	22	17.05	
Smoking status									0.061
No	1266	93.57	149	93.71	382	94.79	184	98.40	
Yes	87	6.43	10	6.29	21	5.21	3	1.60	
Body mass index (kg/m^2^)									0.021
20-25	214	36.58	28	35.90	63	36	18	23.68	
< 20	30	5.13	3	3.85	10	5.71	4	5.26	
25-30	253	43.25	35	44.87	68	38.86	28	36.84	
> 30	88	15.04	12	15.38	34	19.43	26	34.21	
Hospital length of stay (day)									0.001
< 5	720	52.98	70	44.03	176	43.35	75	39.89	
5-10	430	31.64	60	37.74	143	35.22	57	30.32	
11-30	209	15.38	29	18.24	87	21.43	56	29.79	
ICU admission									0.001
No	1002	77.98	106	68.83	278	70.92	118	64.13	
Yes	283	22.02	48	31.17	114	29.08	66	35.87	

 The number (%) of the four study groups and general characteristics stratified by endpoint (lived vs. dead) on the 30th day as well as crude ORs are presented in Table 2. Mortality rates among controls, diabetes, hypertension and hypertension and diabetes were 12.68%, 15.72%, 20.74% and 26.74%, respectively. The odds of death in hypertension alone group and comorbidities hypertension and diabetes group were 1.80 and 2.51 times of controls, respectively (*P* < 0.001). The number (%) of death among patients aged 50 and over (21.88%) than in those under 50 (4.51%) with OR (95% CI) of 5.93 (4.10, 8.56). Unemployed and single patients had greater odds of death within 30 days (*P* < 0.015). Compared to patients with BMI of 20 to 25, those with BMI of less than 20 had OR of 2.29 (*P* = 0.015). With increasing length of stay in hospital the odds of death within 30 days is increased. As expected, patients with ICU admission had greater the odds of death.

**Table 2:** The effect of the study groups, general characteristics and hospital course events on 30-day mortality, univariate logistic regression analysis

**Variables**	**Lived** **(N=1777)**		**Dead** **(N=331)**		**OR (95% CI)**	***P *** **value**
	**Number**	**Percent**	**Number**	**Percent**		
Groups						
Controls	1185	87.32	172	12.68	1.00	
Diabetes	134	84.28	25	15.72	1.28 (0.81, 2.02)	0.281
Hypertension	321	79.26	84	20.74	1.80 (1.35, 2.40)	0.001
Hypertension + diabetes	137	73.26	50	26.74	2.51 (1.75, 3.60)	0.001
Gender						
Female	825	85.14	144	14.86	1.00	
Male	952	83.58	187	16.42	1.12 (0.88, 1.42)	0.328
Age (y)						
< 50	720	95.49	34	4.51	1.00	
≥ 50	1053	78.12	295	21.88	5.93 (4.10, 8.56)	0.001
Location						
City	1485	84.23	278	15.77	1.00	
Village	291	84.59	53	15.41	0.97 (0.70, 1.34)	0.866
Marital status						
Married	1565	85.24	271	14.76	1.00	
Single, divorced, widow	212	78.23	59	21.77	1.60 (1.17, 2.20)	0.003
Occupation						
Employed	681	79.37	177	20.63	1.00	
Unemployed	418	87.82	58	12.18	0.53 (0.38, 0.73)	0.001
Smoking status						
No	1670	84.47	307	15.53	1.00	
Yes	98	80.99	23	19.01	1.27 (0.80, 2.04)	0.309
Body mass index (kg/m^2^)						
20-25	262	81.11	61	18.89	1.00	
< 20	30	65.22	16	34.78	2.29 (1.17, 4.46)	0.015
25-30	322	84.29	60	15.71	0.80 (0.54, 1.18)	0.265
> 30	140	87.50	20	12.50	0.61 (0.35, 1.05)	0.079
Hospital length of stay (day)						
< 5	920	88.46	120	11.54	1.00	
5-10	600	87.21	88	12.79	1.12 (0.83, 1.50)	0.434
11-30	257	67.63	123	32.37	3.67 (2.75, 4.88)	0.001
ICU admission						
No	1452	96.80	48	3.20	1.00	
Yes	229	44.81	282	55.19	37.25 (26.61, 52.14)	0.001

 The results of multivariable analysis are shown in Table 3. Based on three first model, hypertension and diabetes comorbidities was associated with 1.58-fold (adjusting for age), 1.73-fold (adjusting for age and sex) and 2.60-fold (adjusting for sex, age, marital status, occupation and BMI) increase in the odds of death within 30 days. In order to evaluate the independent effects of hypertension alone and in comorbidity with diabetes the models was adjusted to two important hospital course events. Compared to controls, the odds of death within 30 days are increased significantly by 68% and 58% among patients with hypertension after adjusting for hospital length of stay and ICU admission, respectively. The adjusted ORs of the outcome of interest for hypertension and diabetes were 2.13 and 1.91 after adjusting for the two aforementioned factors.

**Table 3:** The effect of the three study groups on death within 30 days compared with control group, multivariable logistic regression analysis

**Adjusted variables**	**Diabetes**		**Hypertension**		**Hypertension+diabetes**	
	**OR (95% CI)**	***P *** **value**	**OR (95% CI)**	***P *** **value**	**OR (95% CI)**	***P *** **value**
Model 1						
Age	0.98 (0.61, 1.56)	0.929	1.11 (0.82, 1.50)	0.477	1.58 (1.06, 2.30)	0.015
Model 2						
Sex and age	1.00 (0.63, 1.61)	0.973	1.18 (0.87, 1.60)	0.290	1.73 (1.18, 2.52)	0.005
Model 3						
Sex, age, marital status, occupation and BMI	0.86 (0.37, 2.01)	0.738	1.44 (0.83, 2.50)	0.195	2.60 (1.29, 5.23)	0.007
Model 4						
Hospital length of stay	1.23 (0.77, 1.96)	0.375	1.68 (1.24, 2.25)	0.001	2.13 (1.46, 3.09)	0.001
Model 5						
ICU admission	0.84 (0.48, 1.47)	0.559	1.58 (1.09, 2.30)	0.015	1.91 (1.18, 3.08)	0.008

## Discussion

 The present study aimed to evaluate the effects hypertension alone and in comorbidity with diabetes on death within 30 days among inpatients with COVID-19 in Hamadan, West of Iran. Our results have demonstrated that hypertension alone and in comorbidity with diabetes may be associated with increased odds of death within 30 days independent of three important factors of older age, higher length of stay in hospital and ICU admission.

 We considered alive at discharge/death within 30 days after admission as the outcome of interest. It argued that 30-day risk-adjusted survival probability can be considered as the quality indicator of hospital care and services.^17^ Based on the observed results, one could think that COVID-19 patients with hypertension and diabetic comorbidity may experience negative adverse outcomes in presence of reducing hospital care quality.

 We did not include laboratory findings in the analysis because previous studies^5,8^ showed that laboratory findings are not significantly associated with mortality in multivariable analysis. We assessed the effect of hypertension alone and in comorbidity with diabetes as well as demographics and clinical characteristics on death within 30 days among inpatients with COVID-19. Our results showed clues for positive association between hypertension alone and in comorbidity with diabetes in multivariable analysis but not for diabetes only.

 Previous studies have demonstrated that hypertension and diabetes are the most common comorbidities among COVID-19 deaths, respectively.^18-20^ The findings of a meta-analysis of observational studies indicated that nearly 30% of COVID-19 deaths are attributed to the 4 risk factors including hypertension, diabetes, smoking and obesity.^6^ In one study by Escobedo-de la Peña et al^21^ the adjusted ORs for effect of hypertension alone and diabetes and hypertension on mortality in inpatients with COVID 19 infection were 1.32 and 1.56, respectively, after adjusting for age, sex, chronic disease comorbidities, smoking and obesity while corresponding figures among outpatients were 1.96 and 2.01, respectively. In another study done by Shi et al,^8^ comorbidity hypertension and diabetes was associated with a three-fold increase of in-hospital mortality among patients with COVID-19. One another study showed ORs lower than 1.3 for independent effect of hypertension and diabetes on mortality.^22^ In a retrospective multicentre cohort study by Zhou et al^5^ nearly 50% of COVID-19 patients had hypertension comorbidity, however, their multivariable analysis provide no significant results for association between hypertension and mortality among inpatients with COVID-19. In the aforementioned study^5^ older age, high Sequential Organ Failure Assessment (SOFA) score, and D-dimer greater than 1 μg/mL are introduced as most determinants of mortality. In another study by Guan et al,^23^ having at least one and ≥ 2 comorbidities of hypertension, diabetes, chronic obstructive pulmonary disease (COPD) and malignant tumor were significantly associated with 1.79 fold and 2.59 fold increase in a composite outcome consisted of admission to an ICU, invasive ventilation or death, respectively. In one study, it has been suggested that the effect of hypertension on mortality is function of antihypertensive treatment,^24^ so that the risk of mortality among hypertensive patients with discontinuation of antihypertensive treatment is 2.17 times of those with antihypertensive treatments after adjusting for confounders. Information about history of medications such as antihypertensive drugs or statins was not available in the medical records. The use of these medications can reduce the risk of cardiovascular and renal diseases. Since these diseases are a risk factor for death related COVID-19, they can be potential confounders for the relationship between hypertension and diabetes and death caused by COVID-19.^25^ To overcome this problem, we considered underlying diseases such as cardiovascular and renal disease as exclusion criteria.

 Our finding showed that effect of comorbidity hypertension and diabetes on death related COVID-19 is stronger than effect of hypertension and diabetes of mortality separately. Compromised innate immune system, exaggerated pro-inflammatory and hypercoagulability states may be pivotal reasons for severity COVID-19 infection among diabetes patients.^26^ Here, hypertension may be a trigger for aforementioned pathophysiological mechanisms among diabetic patients with COVID-19 infection.

 The present study has some limitations: First, some of registered information of patients was gathered via self- reporting and therefore may prone to information bias. Second, missing data for some variables e.g. BMI may threaten the validity of the observed results, Third, it is necessary the death cases after discharging and readmission to be included in the analysis that we have not access to the such cases. Forth, limited cases and sample size for diabetes group can be probable reason for non-significant results for effect of diabetes on mortality in our study and finally, in this study we could not assess the effects of medications of hypertension and diabetes as well as duration of these comorbidities on the study outcome.

## Conclusion

 Hypertension alone and in comorbidity with diabetes is independently associated with death within 30 days among inpatients with COVID-19, holding other well-known determinants of mortality constant.

**Highlights**

- Over one third of COVID-19 inpatients (35.6%) had one or two of hypertension and diabetes comorbidity
- Risk of death within 30 days was 26.7% among COVID-19 inpatients with hypertension and diabetes
- Hypertension alone and in comorbidity with diabetes may be independently associated with odds of death

## Acknowledgments

 The authors of the article consider it necessary to express their gratitude for the financial support of the Vice-Chancellor of Research and Technology from Hamadan University of Medical Sciences (project code: 140105253740 and ethics code: IR.UMSHA.REC.1401.420).

## Conflict of interest

 The present study has no conflict of interest for the authors.
